# Endotyping COPD: hypoxia-inducible factor-2 as a molecular “switch” between the vascular and airway phenotypes?

**DOI:** 10.1183/16000617.0173-2022

**Published:** 2023-01-11

**Authors:** Oleh Myronenko, Vasile Foris, Slaven Crnkovic, Andrea Olschewski, Sonia Rocha, Mark R. Nicolls, Horst Olschewski

**Affiliations:** 1 Division of Pulmonology, Department of Internal Medicine, Medical University of Graz, Graz, Austria; 2 Ludwig Boltzmann Institute for Lung Vascular Research, Graz, Austria; 3 Division of Physiology, Otto Loewi Research Center, Medical University of Graz, Graz, Austria; 4 Department of Anaesthesiology and Intensive Care Medicine, Medical University of Graz, Graz, Austria; 5 Department of Molecular Physiology and Cell Signalling, Institute of Systems, Molecular, and Integrative Biology, University of Liverpool, Liverpool, UK; 6 Division of Pulmonary, Allergy and Critical Care Medicine, Department of Medicine, Stanford University, Stanford, CA, USA

## Abstract

COPD is a heterogeneous disease with multiple clinical phenotypes. COPD endotypes can be determined by different expressions of hypoxia-inducible factors (HIFs), which, in combination with individual susceptibility and environmental factors, may cause predominant airway or vascular changes in the lung. The pulmonary vascular phenotype is relatively rare among COPD patients and characterised by out-of-proportion pulmonary hypertension (PH) and low diffusing capacity of the lung for carbon monoxide, but only mild-to-moderate airway obstruction. Its histologic feature, severe remodelling of the small pulmonary arteries, can be mediated by HIF-2 overexpression in experimental PH models. HIF-2 is not only involved in the vascular remodelling but also in the parenchyma destruction. Endothelial cells from human emphysema lungs express reduced HIF-2α levels, and the deletion of pulmonary endothelial *Hif-2*α leads to emphysema in mice. This means that both upregulation and downregulation of HIF-2 have adverse effects and that HIF-2 may represent a molecular “switch” between the development of the vascular and airway phenotypes in COPD. The mechanisms of HIF-2 dysregulation in the lung are only partly understood. HIF-2 levels may be controlled by NAD(P)H oxidases *via* iron- and redox-dependent mechanisms. A better understanding of these mechanisms may lead to the development of new therapeutic targets.

## Abstract 2

**Upregulation of endothelial HIF-2 may represent a new mechanism of the vascular phenotype in COPD, whereas downregulation of HIF-2 may cause damage of airways and lung parenchyma. HIF-2 may provide an important tool to achieve precision medicine in COPD.** https://bit.ly/3UByVmR

## Introduction

COPD is one of the major causes of chronic morbidity and one of the top three causes of death worldwide [1]. Due to an aging population and continued exposure to risk factors (*e.g.* cigarette smoke and air pollution), the medical and social burdens of COPD are progressively increasing. Being very divergent clinically, COPD is also heterogeneous with respect to the severity of airway, pulmonary parenchyma, lung vessel and systemic vasculature involvement. Therefore, a better understanding of the pathobiology of the disease is crucial for developing novel therapeutic approaches targeting key molecular “hubs” in different lung cells.

Pulmonary vascular involvement, eventually resulting in pulmonary hypertension (PH), is a typical feature of COPD. PH due to COPD (COPD-PH) occurs in a significant proportion of patients, may lead to right heart failure and is characterised by a very poor prognosis [2]. Most patients with COPD-PH belong to group 3 PH (associated with lung diseases and/or hypoxia) [3]. A subgroup of COPD patients present with “out-of-proportion” (severe) pre-capillary PH, significantly reduced diffusion capacity of the lung (*D*_L__CO_) and relatively low oxygen and carbon dioxide partial pressures in the systemic blood, but only moderate airflow limitation. This specific pattern has been proposed to represent the “pulmonary vascular phenotype” [4].

The pathologic mechanisms leading to pulmonary vascular alterations in COPD are complex and include lung hyperinflation due to emphysema, left ventricular failure, increased pulmonary vascular stiffness, and vasoconstriction. The latter, on the one hand, is driven by direct effects of tobacco smoke on the vascular wall with the development of chronic smoking-induced endothelial dysfunction and, on the other hand, by hypoxic pulmonary vasoconstriction (HPV) with remodelling of the small pulmonary arteries [5]. HPV is a specific compensatory response to alveolar hypoxia aiming at redistributing blood flow from the less oxygenated alveoli to the better-oxygenated ones. However, in chronic lung diseases, this physiological response may result in PH. The loss of pulmonary capillaries (at least, radiographically) and small arterioles (distal pruning) with or without emphysema may also contribute to the development of PH, and a decreased *D*_L__CO_ may be an indicator for this particular pathology [6].

Hypoxia-inducible factors (HIFs) play not only a pivotal role in the pathogenesis of COPD-PH, but also in physiologic lung development, including angiogenesis and repair [7, 8]. In this review, we focus on HIF-related mechanisms in COPD and COPD-PH, with an emphasis on the possible role of HIF-2α in the structural lung cells for the pulmonary vascular phenotype. We hope this will contribute to a better understanding of COPD pathogenesis and the development of new biomarkers and therapeutic targets.

## Methods

In this review, we aimed to combine the recent achievements in lung cell phenotyping and interactions, based on state-of-art transcriptomic methods, and recent advances in the understanding of hypoxic signalling in the lung in order to elucidate possible molecular determinants of the vascular phenotype in COPD. We relied on our own experience and the published literature. We performed searches of articles published in PubMed (https://pubmed.ncbi.nlm.nih.gov/) up to 31 July 2022 in the English language using the search terms (including MeSH and free-text search terms) “vascular phenotype”, “vascular remodeling”, “hypoxic signaling”, “hypoxia-inducible factors”, “HIF-2” AND “COPD” OR “LUNG”. We assessed the eligibility of the identified articles by screening the titles and abstracts and thus narrowed down the number of articles used for the review.

## Heterogeneity and complexity of COPD

COPD is a conventional umbrella term that, according to Global Initiative for Chronic Obstructive Pulmonary Disease (GOLD), covers different pathological conditions characterised by persistent respiratory symptoms and airflow limitation due to airway and/or alveolar abnormalities usually caused by exposure to noxious particles/gases and influenced by host factors including genetic predisposition and abnormal lung development [9]. A time-dependent interaction between environmental factors (exposome) and genetic predisposition define an individual's susceptibility and the predominant involvement of different cell populations (epithelial, endothelial and/or immune) in the lung, thus leading to different pathologic traits and the development of particular endo- and phenotypes.

While cigarette smoking is considered a major environmental factor associated with 90% of deaths from COPD in developed countries, the other risk factors that determine the development of particular phenotypes remain poorly understood [10]. Recently, genome-wide association studies have identified many candidate genes for disease development, including, for instance, HO-1 (hemoxygenase-1), IREB2 (iron regulatory binding protein-2), HHIP (hedgehog-interacting protein), RAGE (receptor for advanced glycosylation end products), CHRNA3/5 (cholinergic nicotine receptor alpha 3/5), VEGFR2/KDR (vascular endothelial growth factor receptor-2), MMP-12 (matrix metalloproteinase-12) and others [11, 12].

Regarding COPD endotyping (molecular characterisation), many pathological mechanisms of the disease have been described, including alterations in the innate and acquired immune responses, enhanced oxidative stress, abnormal tissue repair, accelerated cellular senescence, impaired apoptosis and defective efferocytosis [13], and, yet, only congenital α_1_-antitrypsin deficiency is considered as an established COPD endotype [14]. According to Petersen *et al*. [15], different COPD endotypes may be derived from different populations of the susceptible lung cells exposed to the environmental factors over time. Following this idea, the cellular targets of the endotypes range from predominant epithelial (airway and parenchyma) to predominant endothelial (pulmonary vasculature) damage. The particular molecular pathways underlying the different susceptibilities of the lung cell populations in the COPD pathogenesis are not fully understood.

## Vascular component of the disease

Pulmonary vascular involvement in COPD has been extensively studied [4, 16, 17]. We will focus on pre-capillary PH, pulmonary vascular disease and the pulmonary vascular phenotype in COPD.

The vascular component of COPD-PH is haemodynamically characterised by a decreased pulmonary vascular compliance and an increased pulmonary vascular resistance (PVR), which may be due to chronic hypoxic vasoconstriction, pulmonary artery remodelling and loss of the microvasculature [18]. Pulmonary microvascular blood flow measured using gadolinium-enhanced magnetic resonance imaging was reduced even in early COPD, including lung regions without emphysematous changes [19]. Pre-capillary PH has been defined as a mean pulmonary arterial pressure (mPAP) >20 mmHg, pulmonary artery wedge pressure ≤15 mmHg and PVR ≥3 Wood units [20]. COPD-PH must be confirmed by right heart catheterisation and there is evidence that PH is present in a substantial proportion of COPD patients with a prevalence between 30% and 70% [18]. mPAP may increase at a rate of 1 mmHg year^−1^; however, with large individual differences [21, 22], and usually manifests at the advanced stages of COPD. Actually, 90% of GOLD stage IV patients have mPAP >20 mmHg [22]. However, only 1–4% of moderate-to-severe COPD patients develop severe PH (mPAP >35 mmHg), partly associated with mild airflow obstruction, reflecting the vascular phenotype [2, 23]. The first analytical identification of this phenotype succeeded, employing a cluster analysis in a French cohort of patients with severe COPD [24].

Based on pathologic studies of lung specimens, pulmonary vascular remodelling in COPD is characterised by narrowing of small arteries (with a diameter <500 μm) due to intimal and medial thickening, as well as neomuscularisation of small arterioles [18]. Pulmonary artery remodelling also includes changes in the adventitia, including infiltration with inflammatory cells [25, 26].

While chronic HPV may lead to medial thickening of the pulmonary arteries and neomuscularisation of small arterioles, with no or minimal intimal remodelling (thus resembling the lesions observed in highlanders) [27], changes in the intima of the pulmonary arteries indicate the development of endothelial dysfunction, with areas of endothelial cell (EC) denudation and intercellular detachment, as well as so-called “neointimal lesions” that encompass the proliferation of cells that possess features of both endothelial and smooth muscle cells (formerly myofibroblasts), with the deposition of elastic and collagen fibres [17, 28]. These complex intimal changes were suggested to be a result of multiple “hits”, including not only hypoxia, but also toxic effects of airborne particles/gases [16]. Therefore, a combination of at least two types of pulmonary vascular remodelling in COPD – HPV-induced and cigarette smoking-related – may underlie the structural alterations in pulmonary arteries [29].

Vanishing capillary syndrome has been suggested as a hypothesis for the development of the vascular phenotype in COPD, explaining severe PH in association with significantly lowered *D*_L__CO_ and mild-to-moderate airway obstruction [30, 31]. Under chronic hypoxia, in contrast to distinctive angiogenesis in the systemic circulation, human lung capillary rarefaction has been described [32]. However, enhanced angiogenesis with increased bronchial wall vascularity was found in the small airways of COPD patients with moderate bronchial obstruction [33]. Therefore, microvascular alterations in COPD may vary in the different vascular beds (bronchial *versus* pulmonary) and depend on the particular phenotype.

Bunel *et al*. [34] found that patients with severe COPD-PH were characterised by more distinctive neomuscularisation of small arterioles and loss of alveolar capillaries, compared to less severe PH. In general, vascular pruning and capillary rarefaction can be explained by vascular regression due to migration and/or apoptosis of ECs [35]. In accordance with the latter, inhibition of EC apoptosis prevented the development of PH in monocrotaline and SU5416 (sugen) animal models [36]. Alternatively, vessel loss may also result from so-called “intussusceptive vascular pruning”, being a consequence of significant blood flow limitation in capillaries and/or downregulation of VEGF signalling in ECs [37, 38]. Therefore, in COPD, severe PH may result from a different pathological mechanism than the pulmonary arterial remodelling in mild-to-moderate PH.

Interestingly, in contrast to the loss of parenchymal blood capillaries, there was an increased lymphatic microvessel density in COPD lung parenchyma [39]. Therefore, dysregulated blood capillary and lymphangiogenesis may represent important components of the vascular remodelling in COPD. For instance, mutations of VEGFR2/KDR were associated with a specific phenotype of idiopathic pulmonary arterial hypertension that is reminiscent of the vascular phenotype in COPD including severe PH and low *D*_L__CO_ [12, 40, 41]. Further studies are required to explore the cellular and molecular mechanisms of vascular remodelling in COPD, taking into account unique features of the lung physiology such as the dual circulation, a particularly oxygen-rich environment and an ever-moving matrix [42].

## HIF as a key molecular “hub” in lung physiology and COPD

HIF is a heterodimeric transcription factor that consists of a constitutively expressed HIF-1β subunit and an O_2_-regulated HIF-α subunit (HIF-1α or HIF-2α): under normoxia, the HIF-α subunit is hydroxylated by PHD (prolyl hydroxylase) and degraded by proteasome *via* the von Hippel–Lindau (VHL) ubiquitylation complex [43]. HIFs play a key role in lung development and homeostasis, including repair and angiogenesis [7, 44]. HIF-1α is expressed and stabilised by hypoxia in more-or-less all lung cells, whereas HIF-2α expression is restricted to the endothelium, alveolar epithelial type 2 cells (AEC2), and several types of bronchial epithelial cells [45]. Interestingly, in the lungs, during fetal development, HIF-1α protein is abundant in the branching epithelium, whereas HIF-2α protein is found in both epithelium and mesenchymal structures that are important for the vessel formation [44]. Also, it is generally believed that HIF-1α drives the early stages of angiogenesis, whereas HIF-2α is required for maturation of the vascular network [46]. Thus, in vascular biology, HIF-2α has been considered a central regulator of physiological and pathological angiogenic phenotypes [8].

*Hif-2*α*^+/–^* mice (haploinsufficient for HIF-2α) are characterised by augmented carotid body sensitivity to hypoxia, irregular breathing and systemic hypertension [47], but they are completely protected from hypoxic PH [48]. Dai *et al*. [49] confirmed this and showed that HIF-2α overexpressing mice developed very severe PH. On the parenchymal level, HIF-2α may play a different role, as recently revealed by Pasupneti *et al*. [50]: human emphysema lung ECs expressed reduced HIF-2α levels and EC-specific deletion of *Hif-2*α in the mouse model led to emphysema development, whereas its overexpression prevented emphysema after SU5416 exposure. Considering this and the fact that HIF-2 controls the main target genes that are involved in the vascular remodelling in PH, HIF-2 may represent a molecular “switch” between the development of the vascular phenotype that is associated with well-ventilated but poorly perfused lung and the nonvascular (airway) phenotypes characterised by poor ventilation–perfusion matching and severe hypoxemia and hypercapnia.

The next sections provide an overview of the main intercellular interactions within lung parenchyma, small pulmonary arteries and small airways, with an emphasis on the role of the HIF system in homeostatic signalling and pathogenesis of the disease.

### Structure and role of HIFs in the alveolar-capillary unit (ACU)

**FIGURE 1:**
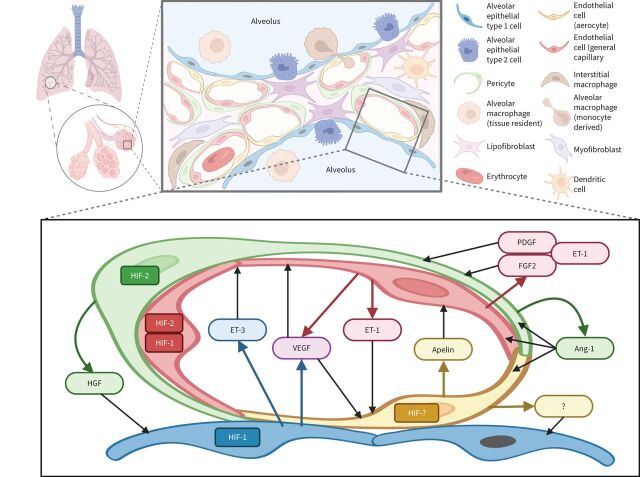
Schematic representation of paracrine interactions within the alveolar-capillary unit of the lung acinus: focus on the microvasculature. An alveolar capillary consists of “respiratory” and “homeostatic” compartments. The first one provides gas exchange and is composed of alveolar epithelial type 1 cells, aerocytes (aCap) and a thin basal membrane between them. The second one consists of closely connected general capillary endothelial cells (gCap) and pericytes that both may contribute to structural stability, regeneration and vascular tone. Crosstalk between gCap and pericytes is mediated by angiopoietin-1 (Ang-1), endothelin (ET)-1, platelet-derived growth factor (PDGF) and fibroblast growth factor-2 (FGF2). Aerocytes are unique microvascular endothelial cells with a large surface designed for effective gas diffusion. They produce apelin that is antagonistic to ET-1 released by gCAP. In addition, a subset of gCap cells represents progenitors for both gCap and aCap. The main pro-survival and mitogenic signals for endothelial cells within the alveolar-capillary unit are vascular endothelial growth factor (VEGF) and Ang-1, produced by alveolar epithelial type 1 cells and pericytes, respectively. Secretion of most of the angiocrine factors is hypoxia-inducible factor (HIF)-dependent: HIF-2α is a predominant form in pericytes and gCAP, while HIF-1α is predominantly expressed in alveolar epithelial type 1 cells. HIF isoforms in aerocytes and factors that may affect alveolar epithelial cells have not been described. HGF: hepatocyte growth factor.

**FIGURE 2:**
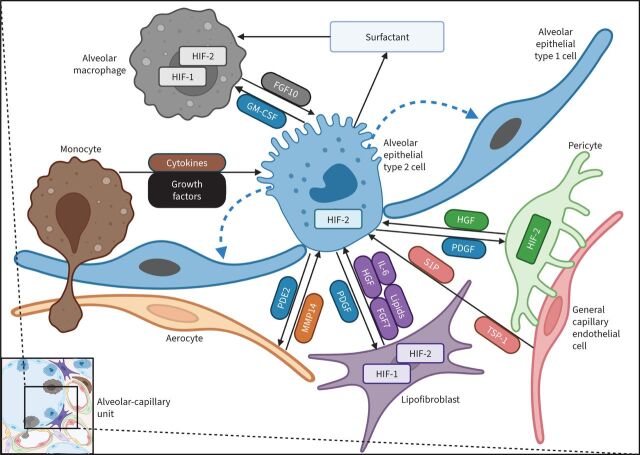
Schematic representation of paracrine interactions within the alveolar-capillary unit of the lung: focus on the alveolar epithelium. Alveolar epithelial type 2 (AEC2) cells play a central role in homeostasis as progenitor cells for alveolar epithelial type 1 cells (blue dashed arrows) and producers of surfactant. AEC2 cells are regulated by multiple paracrine interactions between neighbouring cells. The main pro-survival signal is mediated by hepatocyte growth factor (HGF), produced by lipofibroblasts (alveolar fibroblasts) and pericytes in a hypoxia-inducible factor (HIF)-2-dependent manner. Additionally, fibroblasts secrete interleukin-6 (IL-6) and fibroblast growth factor (FGF)-7. In turn, AEC2 cells produce platelet-derived growth factor (PDGF) that regulates fibroblast and pericyte activity. Capillary endothelial cells secrete matrix metalloproteinase-14 (MMP-14), which increases the bioavailability of the cryptic ligands for the epidermal growth factor receptor on AEC2, thus promoting regenerative alveologenesis. On the other hand, AEC2 cells constitutively release prostaglandin E_2_ (PGE_2_), promoting endothelial barrier integrity. Alveolar macrophages and bone marrow-derived monocytes can be the source of additional factors affecting AEC2 function and proliferative activity (cytokines, growth factors). In turn, AEC2 cells regulate the pool of alveolar macrophages by granulocyte–monocyte colony stimulating factor (GM-CSF). HIF-2α is the predominant isoform in AEC2 and, probably, pericytes, while fibroblasts express both HIF-1α and HIF-2α. S1P: sphingosine-1-posphate; TSP-1: thrombospondin-1.

To date, transcriptomic methods have revealed 58 molecular cell types in human healthy lung: 15 epithelial, nine endothelial, nine stromal and 25 immune populations [51]. At the level of the ACU, an intimate cooperation of lung cells and a precise regulation of their interactions is essential for oxygen transport into the arterial blood. At least 10 different cell types are involved in the spatial and temporal organisation of the ACU (figures 1 and 2). Mapping of the ligand-receptor signalling interactions within the unit predicts hundreds of communications among neighbouring cells [51].

#### Microvascular ECs

HIFs are crucial factors for ECs. The latter play a pivotal role in lung antenatal development, post-natal homeostasis and tissue regeneration by releasing paracrine (“angiocrine”) cytokines, growth factors and metabolites [52]. Transcriptomic studies have discovered a mosaic pattern and stable functional compartmentalisation of two distinct molecular phenotypes of microvascular ECs within the ACU: general capillary (gCap) ECs and so-called “aerocyte” capillary (aCap) ECs [53, 54]. Compared to aCap ECs, gCap ECs have different transcription profiles and functions producing vasoactive substances (endothelin (ET)-1, NO and prostaglandin I_2_) and closely interacting with pericytes [55]. In addition, gCap ECs are considered to be progenitor cells for all microvascular ECs in the lung [56]. Furthermore, network analysis predicted gCap ECs to be a major source of CXCL-12 (C-X-C motif chemokine ligand-12) in the ACU of COPD lungs [57]. CXCL-12 is controlled by HIFs and mediates the recruitment of bone marrow derived angiogenic cells [58]. In turn, aCap ECs are specialised for gas exchange, having a large surface area which is five times greater than that of gCap ECs [53, 59].

Two types of pulmonary microvascular ECs have reciprocal regulation: aCap ECs produce apelin that serves as a ligand for the apelin receptor displayed by gCap ECs; in turn, gCap ECs are a source of ET-1 and VEGF, which both act through cognate receptors on aCap ECs (endothelin receptor B (ET_B_) and VEGFR2/KDR, respectively), thus forming a feedback loop in vascular tone regulation [60]. Apelin expression is controlled by HIF-1α and, in chronic hypoxia, apelin-null mice developed severe PH with loss of pulmonary microvasculature [61]. In turn, HIF-2α may play a specific role in vessel integrity by regulating expression of VEGF (alongside with HIF-1α), VEGF receptor-1 (Flt-1), VEGFR2 and Tie2 (tyrosine kinase receptor for angiopoietins) in ECs [62, 63]. Being one of the leading survival factors for ECs, VEGF also regulates antenatal alveologenesis and post-natal maintenance of the alveolar structure [64]. Alveolar epithelial type (AEC) 1 but not AEC2 cells have recently been recognised as a near-exclusive source of VEGF during the late stages of lung development and repair [65]. Despite HIF-dependent upregulation of VEGF expression in chronic hypoxia, its levels were reduced in the epithelial lining fluid from patients with emphysema [66, 67]. The latter corresponds with decreased levels of HIF-2α in severe COPD. However, contrary to emphysema, VEGF levels were increased in the sputum of COPD patients with predominant bronchitis [68], reflecting different phenotypes.

Single-cell connectomic analysis revealed that microvascular ECs received signals not only from neighbouring cells in the alveolar niche, but also from remote ones. For instance, both types of microvascular ECs expressed cognate receptors for calcitonin gene-related peptide (CGRP), which is mainly excreted by pulmonary neuroendocrine cells (PNECs) in the airways [54, 69]. CGRP is a potent pulmonary vasodilator neuropeptide, but its level was increased in rats with hypoxia-induced PH, probably reflecting a compensatory upregulation [70]. In addition, CGRP promoted angiogenesis *via* regulation of the HIF-1α/VEGF axis in ECs [71].

#### Pericytes

Pericytes regulate lung tissue growth and homeostasis *via* the coordination of alveolar epithelial and ECs, mainly through paracrine release of hepatocyte growth factor (HGF) and angiopoetin-1 (figure 1) [72]. In severe COPD, uncoupling of pericytes from the microvasculature, as well as their migration towards the larger pulmonary arteries, are important pathogenic factors of emphysema and PH [73]. Interestingly, knockout of prolyl hydroxylase-2 (PHD2) causing an increase of HIF-2α in pulmonary artery ECs increased pericyte recruitment and muscularisation of small arterioles [74]. In turn, pericyte uncoupling from ECs may be facilitated by angiopoietin-2, which is upregulated in ECs by hypoxia during COPD exacerbations [75].

In mice treated with lipopolysaccharide, knockout of sirtuin-3 resulted in a significant reduction of pericyte/EC coverage due to upregulation of angiopoietin-2, but downregulation of HIF-2α/Notch3 and Tie-2 expression [76]. Another animal model confirmed the development of severe PH with pulmonary pericyte differentiation into contractile cells and subsequent muscularisation of distal arterioles after SU5416 exposure followed by 3 weeks of hypoxia [77]. Later, Yuan *et al*. [78] identified Wnt5a (wingless/integrated-5) as a key regulator of pulmonary pericyte/EC interaction and showed that reduced production of Wnt5 by microvascular ECs resulted in decreased migration and polarisation of pericytes towards ECs, resulting in the development of persistent PH in mice. Intriguingly, HIF-2α drove *Wnt5a* expression in multiple duodenal organoid models [79], but it is not known if the same mechanism is relevant for the pulmonary microvascular ECs.

Reduced expression of HGF associated with fewer ECs and pericytes was noted in the lungs of *Hif-2*α-deficient mice and in the lungs from COPD patients, suggesting HIF-2-dependent pro-survival signalling between ECs, pericytes and alveolar epithelial cells [50, 66].

#### Alveolar epithelial cells

Being thin and flat, covering up to 95% of the gas exchange surface, AEC1 cells form intimate associations with aCap ECs, AEC2 cells and mesenchymal cells [65]. AEC1 cells are the source of ligands not only for VEGF, but also for Wnt (wingless/integrated-1), Shh (Sonic hedgehog) and PDGF (platelet-derived growth factor) pathways. This makes AEC1 cells an important hub of intercellular communications within the ACU in lung morphogenesis and homeostasis [80]. AEC1 cells may also be an important source of ET-3 that, being a selective ligand for ET_B_ on ECs, causes vasodilation and has antiapoptotic effects [81].

AEC2 cells play a central role for surfactant production and as progenitor cells for AEC1 cells (figure 2) [57, 82, 83]. Interestingly, HIF-2α contributes to AEC2 maturation during antenatal development encoding important proteins involved in phospholipid metabolism and surfactant production [84]. Self-renewal of AEC2 cells is controlled by ECs *via* the MMP-14/epidermal growth factor receptor pathway [85], and by intimately connected platelet-derived growth factor receptor α-positive fibroblasts *via* bone morphogenetic protein (BMP), fibroblast growth factor (FGF) 7 and Wnt paracrine signalling [56, 82, 86]. Differentiation of AEC2 cells into AEC1 cells is regulated by sphingosine-1-phosphate (S1P) released by ECs [87]. In addition to its general growth-like factor properties, S1P may act as a canonical activator of HIF-2α expression (*via* the Akt/mammalian target of rapamycin (mTOR) pathway), as it was shown in multiple cancer cell lines [88].

It is worth mentioning that in the human lung, respiratory airway secretory (RAS) cells in the respiratory bronchioles may also act as progenitors for AEC2 cells. RAS cell differentiation into AEC2 cells is regulated by Notch and Wnt signalling that is impaired in COPD [89]. The role of HIFs in RAS cells is not understood.

#### Immune cells

Chronic inflammation plays a pivotal role in both COPD and hypoxic PH [90]. In turn, HIF pathways in multiple immune cell types are context-specific and can significantly affect inflammatory process [91, 92]. The role of leukocyte-specific HIF isoforms in pulmonary inflammation has been investigated [93, 94], suggesting that HIF-2α regulates essential inflammatory functions of immune cells [95].

In murine macrophages, HIF-2α regulated the expression of pro-inflammatory cytokines *in vitro* [96], arginase [97] and polarisation into the M2 phenotype [98], although the latter was not observed in the model of aseptic tissue damage [99]. It is unclear whether HIF-2α dysregulation contributes to alveolar macrophage dysfunction in COPD. Expression of HIF-2α in blood neutrophils was elevated in patients with chronic inflammatory diseases and, in murine neutrophils, HIF-2α appeared to increase cell survival, thus promoting persistent neutrophilic inflammation [100]. In natural killer (NK) cells, HIF-2 limits cellular cytotoxicity [101]. In dendritic cells, HIF-1 has an anti-inflammatory function, while little is known about the role of HIF-2 [91]. The role of HIF-2 in adaptive immunity is yet to be fully elucidated. To date, it was shown that HIF-2α enhanced T-helper cell 9 development, inhibited regulatory T-cell induction [102] and played a substantial role in antigen-specific IgM expression by B-cells [103].

### Role of HIFs in small pulmonary arteries

In all vessels, HIF signalling has a strong impact on vascular wall permeability, angiogenesis and repair after injury, although each vascular bed may respond differently due to distinct differences between HIF-1α and HIF-2α expression [104]. In animal models, endothelial-specific deletion of HIF-2α increased lung vascular permeability in acute lung injury and activation of HIF-2α by inhibition of PHD2 suppressed vascular leakiness [105]. Interestingly, knockout of lymphatic endothelial *Hif-2*α also led to increased lymphatic leakage and lymphedema due to loss of Tie2 tonic activation [106].

**FIGURE 3:**
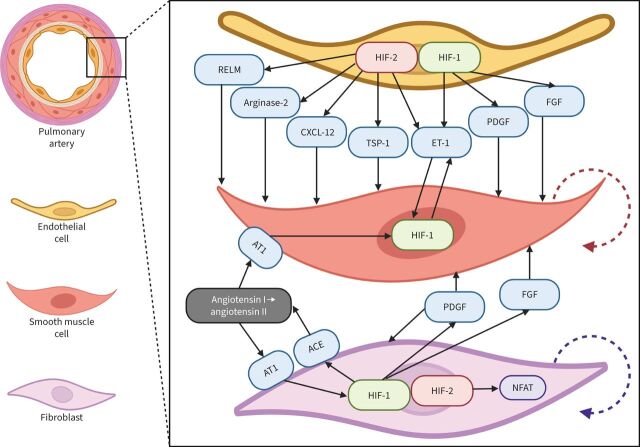
Schematic representation of the hypoxia-inducible factor (HIF)-dependent mechanisms of pulmonary hypertension: focus on small pulmonary arteries. In endothelial cells (ECs), chronic hypoxia causes predominant activation of HIF-2α and subsequent production of vasoconstrictive and mitogenic factors. In turn, HIF-1α is the predominant factor in the smooth muscle cells (SMCs). Among HIF-2α target genes in ECs, C-X-C motif chemokine ligand-12 (CXCL-12), arginase-2, thrombospondin-1 (TSP-1), resistin-like molecule (RELM) and endothelin-1 (ET-1) play important roles in vascular remodelling. CXCL-12 stimulates proliferation (red dashed arrow) and expansion of SMCs and acts as a chemoattractant for immune cells. Arginase-2 limits NO bioavailability causing vasoconstriction. TSP-1 suppresses both the NO and the vascular endothelial growth factor signalling pathways (antiangiogenic effect) and activates transforming growth factor-β (pro-fibrotic effect). RELM also stimulates the proliferation of SMCs. ET-1 is a strong vasoconstrictor and activator of HIF-1α expression in SMCs (feedforward loop). Platelet-derived growth factor (PDGF) and fibroblast growth factor (FGF) are controlled by HIF-1α and secreted by adventitial fibroblasts, in which activation of HIF-1α induces expression of angiotensin-converting enzyme (ACE) that, in turn, causes angiotensin receptor-1 (AT1) stimulation with angiotensin II and subsequent additional upregulation of HIF-1α expression (positive feedback loop). Simultaneously, in fibroblasts, HIF-2α activates nuclear factor of activated T-lymphocytes (NFAT), stimulating fibroblast proliferation (violet dashed arrow) contributing to perivascular fibrosis.

HIFs control the expression of the main target genes that play a key role in the vascular remodelling in PH (figure 3) [107]. For instance, mutations that stabilise HIF-2α protein lead to hereditary erythrocytosis and elevated mPAP even under normoxic conditions [108]. In multiple animal experiments, hypoxic activation of endothelial HIF-2α initiated vascular cell proliferation and recruitment of inflammatory cells in pulmonary arteries in the early stages of PH and, conversely, knockdown of *Hif-2*α or inhibition of HIF-2α significantly reduced the development of hypoxia-induced PH [109]. Previously, beneficial effects of HIF-2α inhibition on the development of PH were shown in different animal models [49]. Although HIF-1α plays an important role for HPV in pulmonary artery smooth muscle cells (PASMCs) and adventitial fibroblasts [110], endothelial HIF-2α appears to be the predominant factor in the pathogenesis of COPD-PH.

Several mechanisms have been proposed to explain the role of endothelial HIF-2α in the development of pulmonary vascular remodelling and PH, including upregulation of ET-1, CXCL-12, arginase-2, thrombospondin-1 (TSP-1), hypoxia-induced mitogenic factor (HIMF)/resistin-like molecule (RELM) and intercellular adhesion molecule 1 (ICAM1), and downregulation of the apelin receptor [111]. Endothelial- and smooth muscle cell-derived CXCL-12 promotes proliferation of PASMCs, increases pericyte coverage, acts as a chemoattractant for the immune cells and stimulates production of HIMF/RELM [112]. Arginase-2 decreases the level of endothelial NO disrupting the NO-dependent mechanism of vascular relaxation [113]. TSP-1 suppresses both NO and VEGF signalling pathways and activates transforming growth factor-β [114]. HIMF/RELM induces the proliferation of smooth muscle cells by triggering the release of HMGB1 (high mobility group box-1) from pulmonary arterial ECs which, in turn, suppresses BMPR2 (bone morphogenetic protein receptor-2) [115]. Several HIF-dependent mechanisms of hypoxic PH have also been reported in PASMCs, where HIF-1α is the main isoform [116].

Regarding the adventitial fibroblasts in PA, HIF-2α but not HIF-1α activates NFAT (nuclear factor of activated T-lymphocytes) signalling, thus promoting fibroblast proliferation [117]. In turn, HIF-1α regulates expression of PDGF, FGF, angiotensin-converting enzyme (ACE) and AT1 (angiotensin receptor-1), thus promoting proliferation of PASMCs [118].

Both HIF-1α and HIF-2α play a role in the recruitment of inflammatory cells in PA but the mechanisms are quite different. While, in PASMCs, HIF-1α is upregulated by HIMF/RELM, promoting activation of monocytes and interleukin-6 secretion in macrophages and fibroblasts [119], HIF-2α orchestrates the transactivation of ICAM1 in ECs, thus promoting recruitment of inflammatory cells [120].

### Role of HIFs in small airways

Cellular and functional heterogeneity of the bronchial epithelium has been described elsewhere [121]. Here, we focus on the HIF signalling in the cells that play an important role in the maintenance and regeneration of the airways.

Airway basal cells are key modulators of lung homeostasis and epithelial regeneration due to their stem cell properties and ability to self-renew and differentiate into most of other cell types including goblet, ciliated and club cells, ionocytes, and PNECs [122]. PNECs are epithelial cells that have many characteristics of neurons, including oxygen sensing and production of CGRP, serotonin and gamma-aminobutyric acid [123]. Hypoxia leads to a proliferation of PNECs due to differentiation of the basal stem cells and this process is driven by stabilisation of HIF-1α, but not HIF-2α [124]. Besides stimulation of type 2 innate lymphoid cells, CGRP is a neuropeptide with angiogenic and vasomotor functions [70].

Club cells secrete protective proteins and proliferate under hypoxic conditions, thus providing protection and maintenance of the bronchial epithelium [125]. In contrast to basal cells and PNECs, the hypoxic response of club cells requires HIF-2α stabilisation that leads to the upregulation of FoxM1 and HIMF/RELM [126]. HIMF/RELM has antiapoptotic effects and is upregulated in bronchial epithelial and AEC2 cells during lung alveolarisation [127]. Intriguingly, under hypoxia, HIMF/RELM may be also produced by pulmonary vascular cells, being a more potent vasoconstrictor than ET-1 or angiotensin II [128].

An additional factor that may play a local cytoprotective role in the lung is erythropoietin; it is expressed at very low levels in bronchial epithelial and AEC2 cells in mice [129]. Considering that HIF-2α is an exclusive regulator of erythropoietin expression in the kidney and liver [130, 131], it is tempting to suggest that the same mechanism of regulation applies in the lung. Thus, HIF-2α upregulation in AEC2 and bronchial epithelial cells would have a beneficial effect on lung function in the case of COPD patients with mild-to-moderate emphysema and/or bronchitis.

## Mechanisms of HIF-2α (dys)regulation in COPD

Cellular adaptation to hypoxia through HIFs has been extensively studied and described elsewhere [132]. Mechanisms of HIF regulation include transcriptional (hormones, cytokines and epigenetics), translational (iron-regulatory proteins, mTORC1/2 and calcitriol) and post-translational (O_2_-dependent and O_2_-independent stabilisation) mechanisms [133]. To date, several distinct post-translational modifications of HIF-1α and HIF-2α have been identified, including hydroxylation, phosphorylation, acetylation, methylation and nitrosylation [134]. In addition, regulatory mechanisms of the HIF pathway *via* ubiquitin and nonubiquitin degradation systems are emerging [135]. Here, we will focus on the pathways that may play important role in up- or downregulation of HIF-2α in COPD.

Currently there is no explanation why pulmonary ECs, AEC2 cells and club cells rely so much more on HIF-2α as compared to HIF-1α in the rest of the lung cells [136]. Interestingly, in the lung tissue, HIF-2α was detectable at higher oxygen levels as compared to other organs, whereas HIF-1α was undetectable even at 6% ambient oxygen concentration [45]. This suggests an important role for nonhypoxic HIF-2α activation and/or stabilisation in the lung. For instance, S1P is an oxygen-independent regulator of HIFs (*via* intracellular iron accumulation and ceramide production) [137]. In addition, inflammatory cytokines, adenosine, intracellular ascorbate and even microbe-derived products (siderophores and short-chain fatty acids) modulate activity of HIFs in both normoxia and hypoxia [138]. Interestingly, decreased *Hif-2*α expression was observed in chronically cigarette smoke exposed mice [139]. However, the particular mechanisms of HIF-2α downregulation after cigarette smoke exposure are not well understood, as well as the presence of a similar effect in humans.

HIF activation is also modulated by intracellular iron. Iron acts as a cofactor for PHDs, and HIF-2α translation is controlled by the iron regulatory proteins-1/2 (IRP1/2) that function as HIF-2α repressors [140]. This explains why *Irp1^−/–^* animals developed PH, having an increased HIF-2α protein level in pulmonary artery ECs, whereas genetic variants near *IREB2* (encoding IRP2) were a risk factor for pulmonary artery enlargement and PH in COPD [141, 142]. This suggests that, in the lung, there are multiple nonhypoxic, but iron-dependent pathways upregulating HIF-2α. In turn, HIFs directly trans-activate several iron-related genes, including the transferrin receptor, heme oxygenase and coeruloplasmin [143], and HIF-2 regulates the expression of frataxin (mitochondrial aconitase chaperone) which assembles iron–sulphur clusters [144]. The mechanisms of abnormal iron homeostasis in COPD have recently been reviewed by Cloonan *et al*. [145].

HIFs can be modified by reactive oxygen species (ROS) in a direct and indirect fashion, but oxidation of the cysteine residues (direct redox effect) is only present in the DNA-binding domain of HIF-2α and not HIF-1α [146]. Whether this HIF-2α feature is cell-type specific or universal is unknown. The indirect effects of ROS are mediated *via* modulation of PHDs, FIH (factor inhibiting HIF), redox-sensitive kinases and phosphatases [147]. In particular, ROS are known to activate the extracellular signal-regulated protein kinase-1/2 (ERK1/2) that phosphorylates HIF-2α, thus controlling its nucleus shuttling and transcriptional activity [148].

**FIGURE 4:**
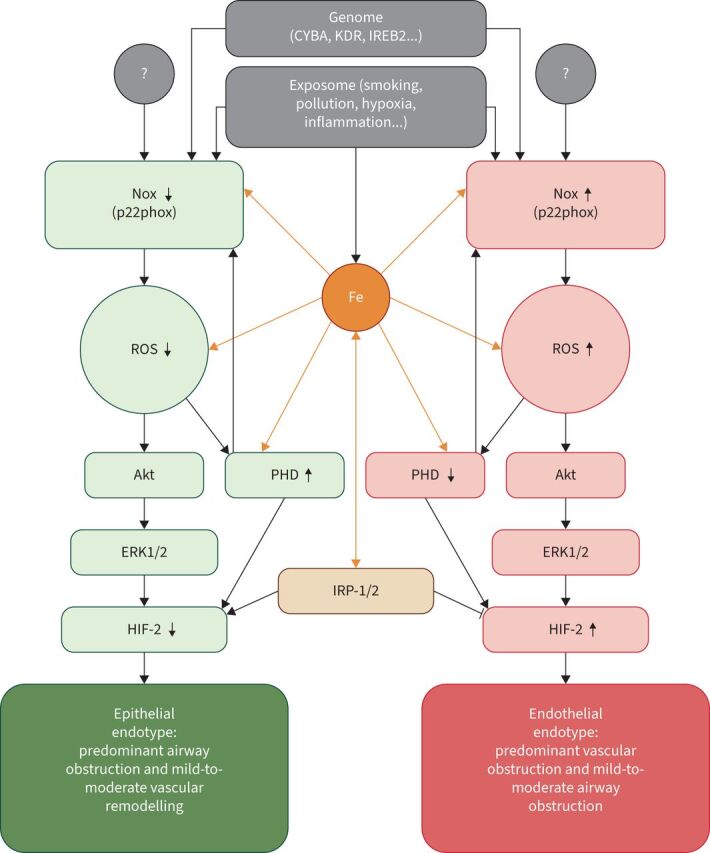
Schematic of the hypothetic mechanism of hypoxia-inducible factor-2α (HIF-2α) (dys)regulation in COPD. NAD(P)H oxidase (Nox) stabilisation (including the p22phox subunit) increases production of reactive oxygen species (ROS) that deactivate prolyl hydroxylase (PHD), but activate the Akt-kinase (Akt)/extracellular signal-regulated kinase-1/2 (ERK1/2) pathway resulting in endothelial HIF-2α upregulation and development of the endothelial endotype. The latter may be associated with the vascular phenotype in COPD. In contrast, reduced expression and/or activity of Nox leads to HIF-2α downregulation and development of the epithelial endotype that may encompass nonvascular phenotypes (bronchitis or emphysema). Since PHD and Nox (p22phox) are iron-dependent enzymes, the intracellular iron level modifies the production of ROS, PHD activity and regulates translation of HIF-2α *via* iron regulatory protein-1/2 (IRP-1/2). Hypoxia, cytokines and environmental factors affect the level and/or activity of Nox. CYBA: gene encoding p22phox; IREB2: gene encoding IRP-2; KDR: gene encoding vascular endothelial growth factor receptor 2.

NAD(P)H oxidases (Nox) are important oxygen sensors and source of ROS in the cells [149]. In COPD, Nox may be an intriguing clue to the possible mechanism of HIF-2α dysregulation and its role as a “switch” between phenotypes. Thus, Nox2-deficient mice developed spontaneous emphysema [150]. Nox subunit NOXO1 (Nox organiser-1) was upregulated in both *Nox2^−/–^* animals and wild-type mice with cigarette smoke-induced emphysema, as well as in human COPD, whereas *Noxo1^−/–^* mice were protected from cigarette smoke-induced emphysema and PH with right heart hypertrophy. However, mice had an increased vascular muscularisation after 8 months of cigarette smoke exposure [151]. Nagaraj *et al*. [152] showed that p22phox, an essential subunit of several Nox [153], was significantly reduced in patients with severe COPD as compared to controls, but was preserved in the subgroup with severely increased mPAP and lowered *D*_L__CO_. Furthermore, downregulation of p22phox expression inhibited Akt phosphorylation and decreased HIF-2α translation [154] while p22phox stabilisation promoted hypoxic PH in mice [155]. In addition, cancer research studies showed that p22phox-dependent Nox1 and Nox4 were essential for HIF-2α expression and activity in VHL-deficient cells [156]. Considering all these facts, we hypothesise that development of the vascular phenotype in COPD may be mediated by stabilisation of several Nox isoforms *via* iron- and redox-dependent factors, controlling endothelial HIF-2α expression and activity (figure 4).

**Points for clinical practice**

- In COPD, upregulation of HIF-2 in the lung may increase the risk of development of the vascular phenotype.
- Cell-specific HIF-2 surrogate readouts (related endothelial, alveolar type 2 epithelial or bronchial epithelial biomarkers) may provide a clue towards better molecular characterisation of the individual COPD patients and their management.

**Questions for future research**

- Will HIF-2 augmentation be beneficial for COPD patients with loss of airway structures?
- Will HIF-2 suppression be beneficial for COPD patients with the pulmonary vascular phenotype?

## Conclusion and future perspectives

The HIF system plays a critical role in the pulmonary development and homeostasis. Cell-specific expression of HIF-2α is predominant in ECs, AEC2 cells and club cells, and mediates unique functions in the lung. In COPD, upregulation of HIF-2 in ECs may lead to predominant pulmonary vascular remodelling and severe PH, but preserved bronchial and parenchymal structures – due to the pro-surviving functions of HIF-2 in AEC2 and bronchial club cells. In contrast, downregulation of HIF-2 in AEC2 and bronchial club cells may lead to parenchymal degeneration and loss of small airways, but more preserved vascular structures due to beneficial effects in pulmonary arterial ECs. These effects of up- or downregulation of HIF-2α may explain, at a molecular level, why different phenotypes of COPD develop.

**FIGURE 5:**
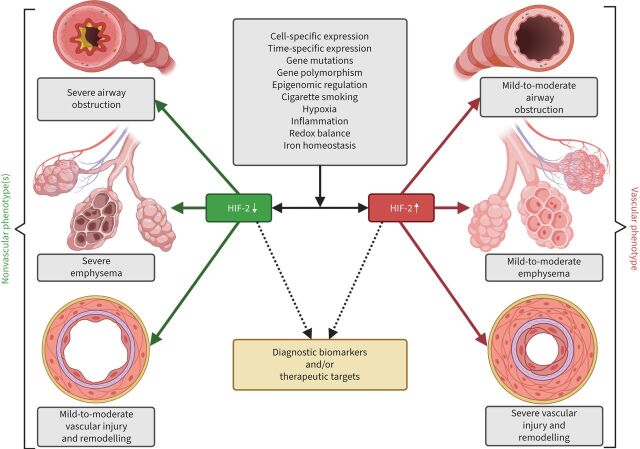
Hypothesis of hypoxia-inducible factor-2 (HIF-2) as a “switch” between the vascular and nonvascular phenotypes in COPD. HIF-2 might represent a molecular “switch” between progression into a vascular or nonvascular phenotype. Under the influence of multiple exogenous and endogenous factors, maladaptive upregulation of HIF-2 in endothelial cells, alveolar type 2 cells and some types of bronchial cells may result in predominant vascular involvement, with pronounced remodelling and severe (out-of-proportion) pulmonary hypertension, but relatively preserved airways and alveolar-capillary units of the lung (the vascular phenotype). Loss of adaptive HIF-2 upregulation (HIF-2 downregulation) in the mentioned cells leads to the loss of alveoli and/or small bronchioles, but mild-to-moderate vascular remodelling and proportionate pulmonary hypertension.

Figure 5 illustrates the putative role of HIF-2 as a “switch” between the vascular and nonvascular phenotypes in COPD. This hypothesis suggests that HIF-2-modulating therapies may affect both the vascular and airway system of the lung. However, it will be important to consider the timing, cell specificity and off-target side effects of such therapies. Nevertheless, potential HIF-2 surrogate readouts may have an important diagnostic meaning in COPD endotyping, establishing the “endotype – biomarker – precision medicine” strategy and identifying new treatable targets.
